# RNA-Seq Using Two Populations Reveals Genes and Alleles Controlling Wood Traits and Growth in *Eucalyptus nitens*

**DOI:** 10.1371/journal.pone.0101104

**Published:** 2014-06-26

**Authors:** Saravanan Thavamanikumar, Simon Southerton, Bala Thumma

**Affiliations:** 1 Department of Forest and Ecosystem Science, University of Melbourne, Creswick, Victoria, Australia; 2 CSIRO Plant Industry, Acton, ACT, Australia; Temasek Life Sciences Laboratory, Singapore

## Abstract

*Eucalyptus nitens* is a perennial forest tree species grown mainly for kraft pulp production in many parts of the world. Kraft pulp yield (KPY) is a key determinant of plantation profitability and increasing the KPY of trees grown in plantations is a major breeding objective. To speed up the breeding process, molecular markers that can predict KPY are desirable. To achieve this goal, we carried out RNA-Seq studies on trees at extremes of KPY in two different trials to identify genes and alleles whose expression correlated with KPY. KPY is positively correlated with growth measured as diameter at breast height (DBH) in both trials. In total, six RNA bulks from two treatments were sequenced on an Illumina HiSeq platform. At 5% false discovery rate level, 3953 transcripts showed differential expression in the same direction in both trials; 2551 (65%) were down-regulated and 1402 (35%) were up-regulated in low KPY samples. The genes up-regulated in low KPY trees were largely involved in biotic and abiotic stress response reflecting the low growth among low KPY trees. Genes down-regulated in low KPY trees mainly belonged to gene categories involved in wood formation and growth. Differential allelic expression was observed in 2103 SNPs (in 1068 genes) and of these 640 SNPs (30%) occurred in 313 unique genes that were also differentially expressed. These SNPs may represent the *cis*-acting regulatory variants that influence total gene expression. In addition we also identified 196 genes which had Ka/Ks ratios greater than 1.5, suggesting that these genes are under positive selection. Candidate genes and alleles identified in this study will provide a valuable resource for future association studies aimed at identifying molecular markers for KPY and growth.

## Introduction

*Eucalyptus nitens* (shining gum) is a perennial forest tree species grown mainly for kraft pulp production (KPY) in many parts of the world [1]. KPY is considered a key determinant of plantation profitability [2] and consequently increased KPY is a major objective of breeding programs [3]. In forest tree species, marker-assisted selection (MAS) is particularly attractive because conventional selection is impeded by long generation times and long delays until the full expression of mature traits [4]. A common feature of most agronomic traits in trees is that they are complex, and likely to be controlled by variation in many genes. Currently, there are two approaches being explored in trees for applying markers in breeding for improvement of complex traits. In the first approach, known as Genomic Selection (GS), large numbers of random markers are used for predicting phenotypes from genotypes [5]. In the second approach, markers potentially controlling the trait occurring within candidate genes are identified using association genetics in candidate genes. These associated markers are then used to predict traits as in GS with random markers [6]. The discovery of high quality candidate genes is therefore a crucial step in the discovery of polymorphisms associated with complex traits such as growth and pulp yield.

Recent developments in sequencing technologies are making it possible to identify large numbers of high quality candidate genes by exploring gene expression at the whole transcriptome level. RNA sequencing (RNA-Seq) uses next generation sequencing technologies to sequence complementary DNA (cDNA), and the resulting sequencing reads are either assembled *de novo* or mapped on to a reference genome, if available. Differential gene expression can be examined by comparing the number of reads mapping to genes in samples derived from different conditions. Such RNA-Seq experiments are new to forest tree species and only a few studies have been published to date [7]–[10]. In addition to the identification of differentially expressed genes, RNA-Seq can also be used to identify differentially expressed alleles [7]. Until recently, microarrays were predominantly used to explore differential expression in large numbers of genes [11]. However RNA-Seq is replacing microarrays to overcome some of the limitations in microarray studies including increased false positives due to hybridization signals [12] particularly from transcripts of low abundance [13]. RNA-Seq is also useful for discovering new transcripts, while microarrays can only detect transcripts that correspond to existing genomic sequence information.

In this study, we used RNA-Seq to identify candidate genes and alleles that may influence wood and growth traits by comparing gene expression in cambial tissue between low and high KPY trees. Cambial tissue is widely used in forest tree species to study patterns of expression of genes involved in wood (xylem) development. KPY is a wood quality trait of forest trees and is influenced by the cellulose and lignin content of xylem. Several studies have shown that virtually all cellulose and lignin biosynthetic genes are expressed in cambial tissue. Therefore cambial tissue is widely used as a key organ to identify genes relating to pulp yield in a number of studies [14]–[17]. In this study, we identified several genes and alleles affecting wood and growth traits which were consistent between two populations. The functional variants showing differential allelic expression identified in this study are useful for future association studies to identify markers for KPY and growth traits.

## Methods

### Plant material and RNA extraction

Plant material from two trials of *E. nitens* at Meunna (−41.08°S, 145.47°E) and Florentine (−42.54°S, 146.51°E) in Tasmania, Australia were used in this study. Meunna and Florentine are approximately 350 kilometres apart and located at an altitude of 297 m and 266 m above mean sea level, respectively. The annual rainfall of Meunna and Florentine are 1007 mm and 1225 mm, respectively. The two trials were established in 1993 to study the performance of 420 *E. nitens* families, each represented by two-tree plots in each of five replicates. Cambial scrapings for RNA extraction were collected from 44 trees, 22 each from high pulp yield and low pulp yield extremes in the Meunna trial (March 2011) and 66 trees, 33 each from high pulp yield and low pulp yield extremes, in the Florentine trial (May 2012). Scrapings were immediately frozen on dry ice then stored at −80°C. Total RNA was isolated from the cambial scrapings following a modified CTAB method as described in [18]. RNA samples were then treated with TURBO DNA-free Kit (Cat No. AM1907, Ambion) to remove contaminating DNA from RNA preparations and to remove the DNAse from the samples. Concentrations of RNA samples were measured using a QUBIT fluorometer and all the samples were normalized to 100 ng/ul. An equimolar concentration of total RNA from trees in each category (high and low pulp yield) was pooled into three bulks of seven to eight trees each in Meunna and 11 trees each in Florentine and quality checked using an Agilent 2100 Bioanalyser. These three bulks from each treatment were used as biological replicates in differential gene expression analyses.

### cDNA Sequencing

In total, six RNA bulks from two treatments (three from high and three from low pulp yield) from each trial were sequenced (paired end) at the Australian Genome Research Facility using the Illumina HiSeq platform (HiSeq 2000). Raw sequence reads were obtained using the Illumina CASAVA pipeline version 1.8.2.

### RNA sequence reads mapping and transcript assembly

Adapter sequences from all raw sequence reads were removed using CLC Genomics Workbench v6.0.4 (CLC Inc, Aarhus, Denmark) and sequence reads having a quality score less than 20 were discarded using the Popoolation package [19]. Quality trimmed sequencing reads from all 6 libraries in each trial were pooled and mapped to the *Eucalyptus grandis* reference genome (http://www.phytozome.net/eucalyptus.php) with TopHat v2.0.9 [20] which uses Bowtie v0.12.7 [21] as an alignment engine. Tophat was run with the default parameters. To determine and exclude ambiguous reads mapping to multiple transcripts we used Tophat’s default option (–g value: 20 multi-hits). Since we do not have a reference genome sequence for *E. nitens*, we used the publicly available *E. grandis* reference genome sequence for mapping the sequencing reads. A binary sequence alignment file (BAM) produced by TopHat and a FASTA file of *E. grandis* genome sequence was used to generate transcript annotations in GTF format using Cufflinks v1.1.0 [22]. Cufflinks was run with default parameters without supplying any annotation file. BEDtools v2.18.1 [23] was used to estimate the counts of reads in individual bulks that are mapping to different gene products in the GTF annotation file using the BAM file from each library. Raw read sequences and the read counts data are deposited in NCBI’s Gene Expression Omnibus and are accessible through GEO series accession number GSE56592.

### Differential gene expression (DGE) analyses

The count files generated using BEDtools for individual bulks were used to find significant differences in transcript abundance between low and high KPY samples using edgeR [24]. EdgeR identifies differentially expressed transcripts based on the assumption that the number of reads produced by each transcript is proportional to its abundance. edgeR measures transcript abundance in counts per million (CPM). As there were three biological replicates each for low and high pulp yield samples in each trial, edgeR observes the differences in the CPMs for each gene across the replicates and uses these variance estimates to calculate the statistical significance (p-values) of observed differential expression. Transcripts with very low expression were filtered before DE analysis based on an expression cut-off of 1 CPM in at least three libraries. For the library sizes in this study, one CPM would correspond to ∼50 read counts for the Florentine trial and ∼70 read counts for the Meunna trial. Benjamini and Hochberg’s algorithm [25] was used to control false discovery rate (FDR) due to multiple testing in differential expression analysis.

A web-based tool High-Throughput GOMiner [26] was used to categorise the differentially expressed genes based on their function. To identify Arabidopsis homologs for gene models predicted from transcriptome mapping, BEDTools was used to extract sequences of all genes from the *E. grandis* reference genome sequence using gene coordinates from the gene annotation (GTF) file produced using the ‘Cufflinks’ package. The extracted gene sequences were BLAST searched with the Arabidopsis protein database. The identified Arabidopsis homologs were used in GO enrichment tests based on Biological processes.

### SNP discovery and differential allelic expression (DAE) analysis

To identify SNPs from the RNA-Seq data, BAM files generated from TopHat were used in SAMtools to produce an mpileup file. Reads from the three biological replicates from each treatment were combined to increase coverage and confidence of the SNP calls. The mpileup file was used in VarScan [27] to call SNPs. The following parameters were used in SNP calling: minimum read depth (50), minimum supporting reads (20), minimum base quality (20), minimum variant allele frequency (0.01), P-value threshold for calling variants (0.05). We used these stringent parameters compared to the less stringent default parameters to avoid false positives in SNP calling. We also tested for differences in the frequency of the alleles at each SNP between low and high pulp yield samples in each trial. A chi-square test was performed to estimate the significance of allele frequency differences.

A GO analysis was conducted using High-Throughput GOMiner to categorise the genes that had differentially expressed alleles based on their function. Separate analysis was performed for genes that showed both DGE and DAE and genes that showed only DAE.

### Identification of genes under positive selection based on Ka/Ks ratios

We used Popoolation to annotate synonymous (SS) and nonsynonymous (NS) substitutions using an mpileup file containing reads merged from all the six bulks from each trial and a coding sequence (CDS) gene annotation file of *E. grandis*. A minimum allele count of 4, minimum coverage of 20 reads, maximum coverage of 2000 reads and a minimum phred quality of 20 was used to identify SNPs. The identified nonsynonymous and synonymous SNPs were used to estimate Ka/Ks ratios (ratio of number of nonsynonymous substitutions per nonsynonymous site to the number of synonymous substitutions per synonymous site). The nonsynonymous and synonymous SNPs were normalized by their respective lengths estimated with the Popoolation package. A constant 1 is added to the number of SNPs to enable comparisons with genes containing no SNPs, as suggested by [28]. Genes with Ka/Ks ratio of more than 1.5 were considered genes under positive selection. We also conducted GO enrichment tests to identify the biological processes associated with the genes showing positive selection signatures.

## Results

### Sequencing output

**Figure 1:**
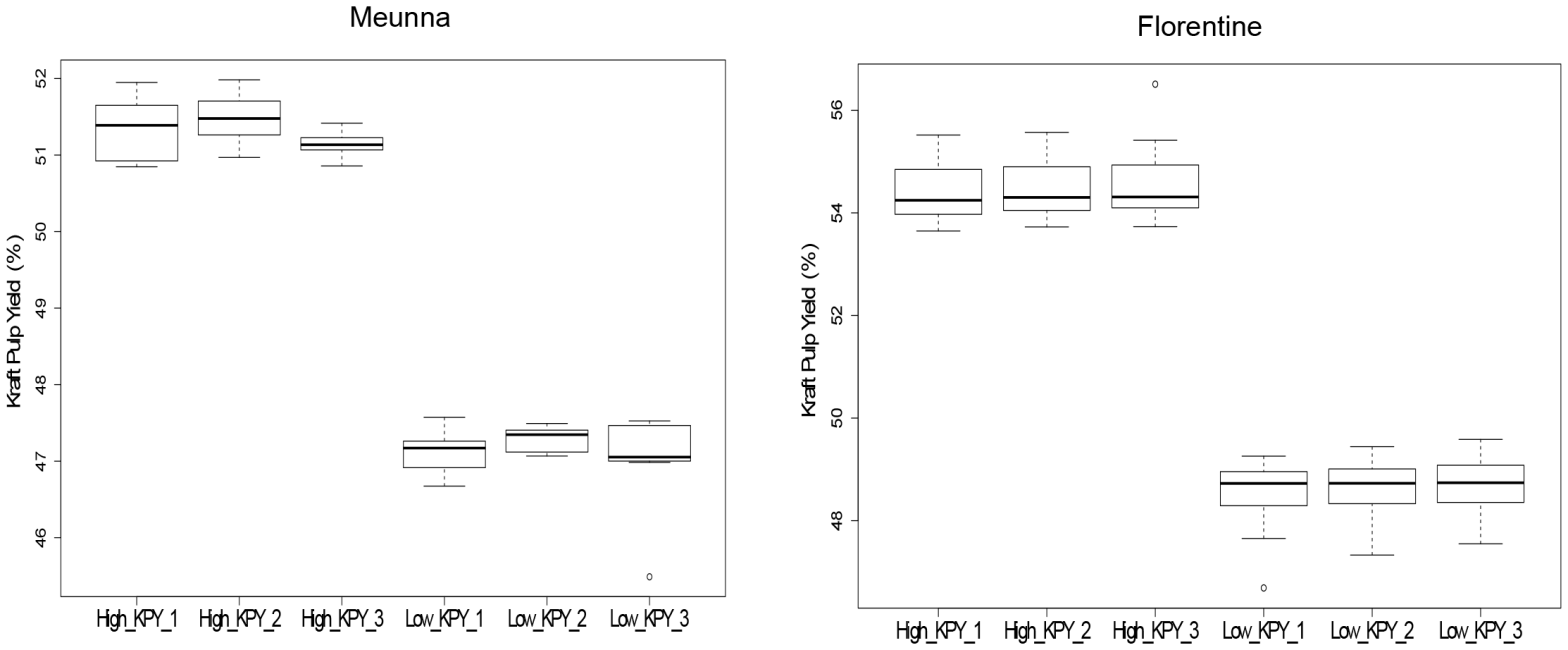
Distribution of Kraft Pulp Yield for samples collected from the Meunna and Florentine trials.

RNA samples of *E. nitens* trees representing the KPY extremes were collected in two trials, Meunna and Florentine. Distributions of pulp yield for the collected samples from both the trials are shown in Fig. 1. A positive correlation between KPY and diameter at breast height (DBH) was observed for samples in both Meunna and Florentine (Figure S1).

From both the Meunna and Florentine trials, six RNA bulk libraries from two treatments (three from high and three from low pulp yield) were paired-end sequenced on one lane of an Illumina HiSeq flowcell. In Meunna this yielded a total of 430 million reads, with individual library yields ranging from 49 to 78 million reads. In Florentine, this yielded a total of 286 million reads, with individual libraries yielding 43 to 53 million reads. These reads were mapped to the *E. grandis* reference genome using Bowtie and TopHat software packages. Sequencing reads from three bulks within a treatment were used as biological replicates in differential gene expression analyses.

### Differential gene expression analysis

To identify the candidate genes controlling KPY and growth traits, we performed differential gene expression (DGE) analysis using edgeR on the *E. nitens* transcripts which had a minimum of one counts per million (CPM) in at least three libraries. The down-regulated genes in low KPY (low DBH) samples are primarily involved in growth and cell wall formation while up-regulated genes in low KPY samples (up-regulated in high KPY samples) are mainly involved in biotic and abiotic stress tolerance reflecting the low growth of the low KPY samples. Several genes putatively involved in wood formation and growth such as alpha and beta-tubulins, calcium dependent protein kinase, cellulose synthases, cellulases, COBRA-like proteins, 4-coumarate:CoA ligase, FASCICLIN-like arabinogalactan protein, MYB domain proteins, protein kinases, SAM-dependant methyltransferases, sucrose synthases, xyloglucan endotransglucosylases were down-regulated in low KPY samples (up-regulated in high KPY samples). On the other hand, biotic and abiotic stress related proteins such as several heat shock proteins, pathogenesis related proteins, senescence related genes, zinc induced facilitator-like proteins and WRKY DNA binding proteins were present among the up-regulated genes in low KPY (low growth) samples.

**Figure 2:**
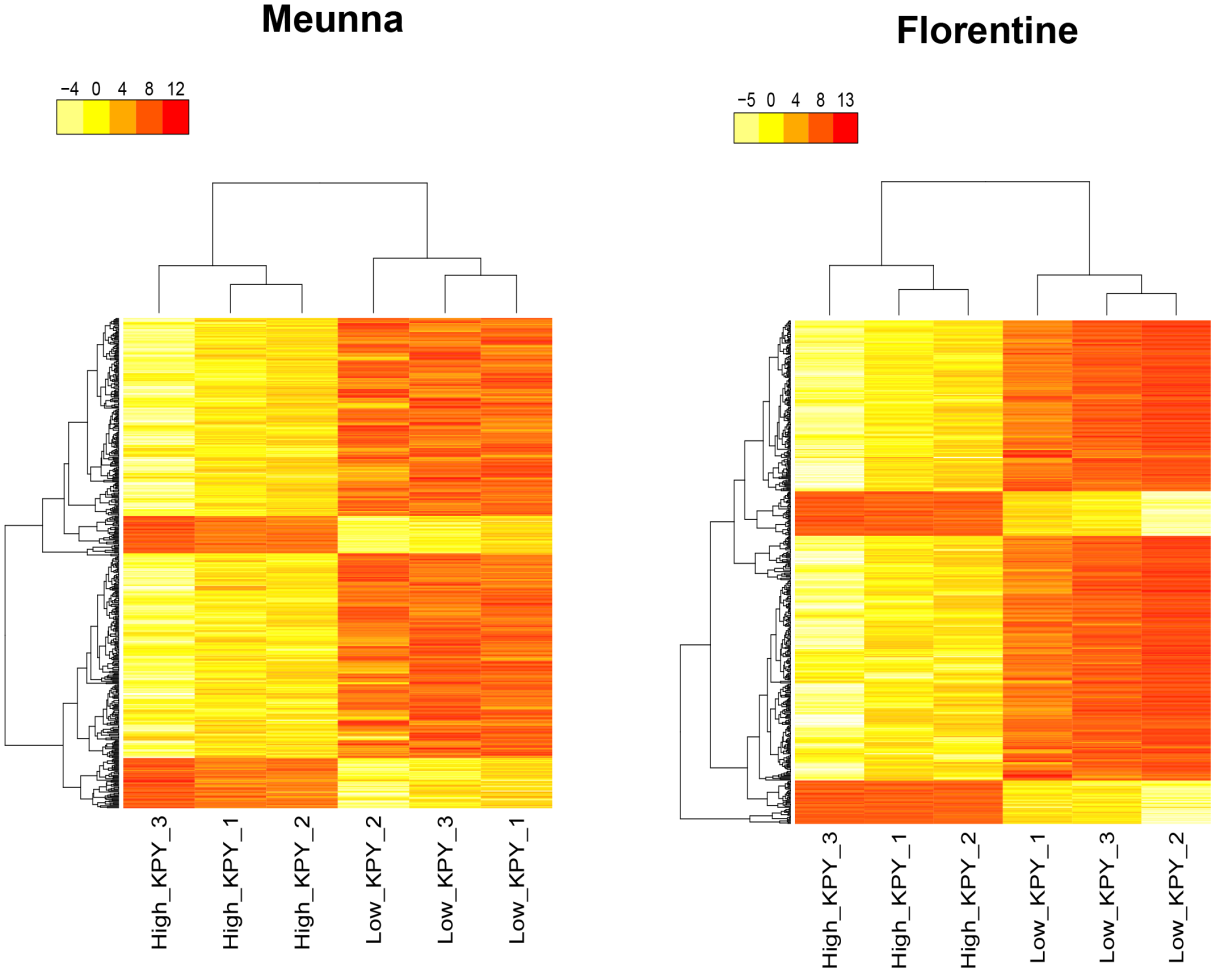
Heatmap of 500 most differentially expressed genes between low and high KPY samples in the Meunna and Florentine trials.

Overall, 32,903 and 30,570 transcripts were predicted in Meunna and Florentine trials, respectively. After filtering for low expression transcripts, 26,279 and 23,917 transcripts from Meunna and Florentine trials were used in DGE analysis. Log2 fold changes between low and high KPY samples ranged from −6.79 to 6.26 in Meunna and from −7.75 to 8.18 in Florentine. To reduce false positives, only transcripts that were differentially expressed at 5% FDR level were declared as DE genes. At 5% FDR level, a total of 6122 and 7240 transcripts (4479 and 5528 unique genes based on *E. grandis* annotations) showed differential expression between low and high KPY samples in Meunna (Table S1) and Florentine (Table S2) respectively. Of these, 3615 (59%) transcripts were down-regulated and 2507 (41%) up-regulated in low KPY samples in Meunna. In Florentine, 3674 (51%) were down-regulated and 3566 (49%) were up-regulated in low KPY samples. Heatmaps were generated for both the trials using log2CPM of the top 500 genes that were differentially expressed in both trials (Fig. 2). Within a treatment (e.g low KPY) gene expression was similar among the three replicates while it was distinct between treatments in both the trials. To determine the relationship between gene expression in Florentine and Meunna the data used for drawing the heatmaps was used to generate dendrograms based on hierarchical clustering (Figure S2). The low and high KPY samples from both the trials were assigned to two separate major groups confirming the similarity between biological replicates within and between trials.

Comparing the DGE between the two trials, 3972 transcripts were significantly differentially expressed in both the trials at 5% FDR level. Of these, only 19 transcripts (0.5%) showed opposite patterns of expression. Of the 3953 transcripts that had gene expression changes in the same direction in both trials, 2551 (65%) were down-regulated and 1402 (35%) were up-regulated in low KPY (low growth) samples (Table S3). Correlation between Log fold changes in Meunna and Florentine for these 3953 transcripts is very high (Figure S3). Differential expression of the top 25 down-regulated genes and top 25 up-regulated genes are shown in Tables 1 and 2, respectively. Transcript gene coordinates and gene identities of all significantly (FDR<0.05) differentially expressed transcripts in both the trials are shown in Table S3.

**Table 1:** Top 25 down-regulated transcripts in low KPY samples.

Gene ID	Meunna		Florentine		TAIR gene annotation
	LogFC	FDR	LogFC	FDR	
Eucgr.E01020	−2.4	7E-10	−2.0	4E-06	ABL interactor-like protein 2
Eucgr.J01011	−2.8	1E-06	−2.0	2E-05	cytochrome P450, family 77, subfamily A, polypeptide 4
Eucgr.I01292	−2.6	4E-06	−2.0	5E-06	Dehydrin family protein
Transcript_23294	−3.3	8E-10	−2.3	1E-07	delta(3), delta(2)-enoyl CoA isomerase 1
Eucgr.E04327	−4.9	2E-11	−2.0	2E-05	DNA glycosylase superfamily protein
Eucgr.F03723	−2.7	4E-06	−2.1	2E-05	expansin 11
Eucgr.E01366	−2.5	9E-08	−2.0	3E-05	FASCICLIN-like arabinogalactan protein 8
Eucgr.J00937	−3.4	2E-05	−2.4	3E-06	FASCICLIN-like arabinogalactan-protein 11
Eucgr.J00938	−3.0	1E-08	−2.2	5E-08	FASCICLIN-like arabinogalactan-protein 12
Eucgr.B02486	−3.0	8E-09	−2.1	8E-06	FASCICLIN-like arabinogalactan-protein 12
Eucgr.C00602	−2.1	2E-05	−2.0	3E-09	GATA transcription factor 12
Eucgr.K03566	−3.3	1E-09	−2.3	4E-06	GDSL-like Lipase/Acylhydrolase superfamily protein
Eucgr.B00543	−2.3	2E-05	−3.5	2E-13	Malectin/receptor-like protein kinase family protein
Eucgr.K01501	−3.3	9E-11	−2.2	5E-06	plasma-membrane associated cation-binding protein 1
Eucgr.J02930	−3.6	3E-12	−2.0	5E-05	profilin 5
Eucgr.H04207	−3.1	2E-06	−2.0	1E-05	Protein of Unknown Function (DUF239)
Eucgr.H04514	−2.9	4E-11	−2.0	1E-06	respiratory burst oxidase homolog B
Eucgr.C00771	−2.6	4E-07	−2.0	5E-07	SAUR-like auxin-responsive protein family
Eucgr.I00074	−2.7	2E-08	−2.0	2E-05	sucrose synthase 2
Eucgr.I00074	−2.7	2E-08	−2.0	2E-05	sucrose synthase 2
Eucgr.H03496	−2.8	7E-08	−2.0	9E-07	sucrose synthase 4
Eucgr.F02183	−2.1	2E-06	−2.0	7E-08	Tubulin/FtsZ family protein
Eucgr.C01361	−2.4	1E-07	−2.1	3E-05	No-Hit
Eucgr.H01054	−3.9	1E-06	−2.8	7E-10	Unknown Protein
Eucgr.H03407	−4.1	5E-08	−2.5	3E-07	Unknown Protein

*E. grandis* gene names are used when the predicted genes are mapped to *E. grandis* gene coordinates otherwise the predicted gene names are used with a prefix “Transcript”.

**Table 2:** Top 25 up-regulated transcripts in low KPY samples.

Gene ID	Meunna		Florentine		TAIR gene annotation
	LogFC	FDR	LogFC	FDR	
Eucgr.C03986	2.9	9E-13	3.9	5E-12	basic leucine zipper 9
Eucgr.I00675	3.0	6E-07	3.5	2E-11	basic leucine-zipper 5
Eucgr.K00864	2.2	8E-08	3.7	3E-15	B-box type zinc finger family protein
Eucgr.J00646	2.2	3E-07	3.2	6E-14	beta glucosidase 11
Eucgr.H04032	2.0	1E-06	2.4	1E-10	cAMP-regulated phosphoprotein 19-related protein
Eucgr.A00523	4.0	5E-15	3.7	4E-14	cytochrome P450, family 716, subfamily A, polypeptide 1
Eucgr.A00523	4.0	5E-15	3.6	2E-13	cytochrome P450, family 716, subfamily A, polypeptide 1
Eucgr.A00523	4.0	5E-15	2.6	5E-11	cytochrome P450, family 716, subfamily A, polypeptide 1
Eucgr.F00146	2.9	3E-06	3.6	6E-13	cytochrome P450, family 81, subfamily D, polypeptide 2
Eucgr.J02333	4.6	5E-13	4.5	2E-13	Galactose oxidase/kelch repeat superfamily protein
Eucgr.K01641	2.1	8E-06	2.5	7E-11	glucose-6-phosphate dehydrogenase 5
Eucgr.A00159	2.6	8E-07	4.0	9E-11	MLP-like protein 423
Eucgr.D00215	3.2	1E-08	2.7	1E-10	multidrug resistance-associated protein 2
Eucgr.I00060	1.9	3E-06	3.8	9E-13	NAC (No Apical Meristem) domain transcriptional regulator superfamily protein
Eucgr.D01888	5.7	4E-09	5.7	2E-17	osmotin 34
Eucgr.A02434	2.4	4E-07	4.3	2E-17	polygalacturonase inhibiting protein 2
Eucgr.C02985	4.6	2E-10	5.2	1E-14	Protein kinase family protein with leucine-rich repeat domain
Eucgr.E02844	2.3	8E-08	3.6	5E-15	receptor-like kinase in flowers 1
Transcript_12193	2.0	2E-06	3.0	5E-15	RNA polymerase subunit beta
Eucgr.F03603	2.5	5E-10	2.6	3E-11	RNA-binding (RRM/RBD/RNP motifs) family protein
Eucgr.I01260	3.1	3E-07	3.3	3E-13	unknown seed protein like 1
Eucgr.I01260	3.1	3E-07	3.3	9E-13	unknown seed protein like 1
Eucgr.F03955	3.6	1E-06	3.4	3E-14	WRKY DNA-binding protein 40
Eucgr.D01937	2.3	2E-08	2.8	4E-11	Unknown Protein
Eucgr.D01937	2.3	2E-08	2.8	1E-10	Unknown Protein

*E. grandis* gene names are used when the predicted genes are mapped to E. grandis gene coordinates otherwise the predicted gene names are used with a prefix “Transcript”.

### Gene Ontology (GO) enrichment analysis of differentially expressed genes

We performed gene ontology (GO) enrichment analyses to functional characterisation of genes showing differential expression. The gene ontology analysis by High-Throughput GoMiner revealed differential enrichment of genes into various biological processes. The genes up-regulated in low KPY (low growth) samples were enriched in biotic and abiotic stress responsive processes. On the other hand, most of the down-regulated genes (up-regulated in high KPY and high growth samples) belonged to gene categories involved in wood formation and growth (Table 3). In Meunna, 57 gene categories were enriched among the genes differentially expressed between low and high KPY samples at the 5% FDR level. Of these, 18 were up-regulated and 39 were down-regulated in the low KPY samples. Thirty nine gene categories were enriched among the genes differentially expressed between low and high KPY samples in Florentine. Of these, five categories were up-regulated and 34 down-regulated in the low KPY samples. Five gene categories were up-regulated and 26 down-regulated in the low KPY samples in both trials, providing more confidence in the enrichment of these gene categories.

**Table 3:** Gene categories enriched among down-regulated and up-regulated genes in low KPY samples.

GO category	Meunna			Florentine		
	Totalgenes	Changedgenes	FDR	Totalgenes	Changedgenes	FDR
**Down-regulated**						
GO:0006793_phosphorus_metabolic_process	337	81	0.00	318	91	0.00
GO:0006796_phosphate_metabolic_process	337	81	0.00	318	91	0.00
GO:0005975_carbohydrate_metabolic_process	201	54	0.00	181	57	0.00
GO:0016310_phosphorylation	316	78	0.00	300	86	0.01
GO:0006468_protein_phosphorylation	307	77	0.00	291	85	0.01
GO:0008361_regulation_of_cell_size	34	14	0.01	33	15	0.01
GO:0016049_cell_growth	34	14	0.01	33	15	0.01
GO:0032535_regulation_of_cellular_component_size	34	14	0.01	33	15	0.01
GO:0090066_regulation_of_anatomical_structure_size	34	14	0.01	33	15	0.01
GO:0033036_macromolecule_localization	137	37	0.01	136	46	0.00
GO:0006464_protein_modification_process	420	93	0.01	393	104	0.00
GO:0009825_multidimensional_cell_growth	9	6	0.01	7	5	0.05
GO:0040007_growth	45	17	0.01	42	17	0.01
GO:0015031_protein_transport	102	29	0.01	100	33	0.02
GO:0045184_establishment_of_protein_localization	102	29	0.01	100	33	0.02
GO:0008104_protein_localization	108	30	0.01	106	34	0.01
GO:0007167_enzyme_linked_receptor_protein_signaling_pathway	27	11	0.02	27	12	0.03
GO:0007169_transmembrane_receptor_protein_tyrosine_kinase_signaling_pathway	27	11	0.02	27	12	0.03
GO:0043412_macromolecule_modification	461	96	0.03	432	107	0.01
GO:0023033_signaling_pathway	121	32	0.03	118	35	0.03
GO:0048869_cellular_developmental_process	56	18	0.03	55	19	0.05
GO:0051234_establishment_of_localization	310	68	0.03	289	77	0.02
GO:0000902_cell_morphogenesis	29	11	0.04	29	12	0.05
GO:0051179_localization	319	69	0.05	298	78	0.01
GO:0006810_transport	309	67	0.05	288	76	0.01
GO:0007264_small_GTPase_mediated_signal_transduction	38	13	0.05	40	15	0.05
**Up-regulated**						
GO:0006950_response_to_stress	385	81	0.00	345	103	0.00
GO:0006952_defense_response	156	48	0.00	128	45	0.00
GO:0050896_response_to_stimulus	592	107	0.00	546	140	0.00
GO:0009628_response_to_abiotic_stimulus	97	32	0.00	178	54	0.02
GO:0006915_apoptosis	83	31	0.00	71	25	0.05

FDR – Fisher’s exact *p* value corrected for multiple comparisons.

### SNP identification and Differential allelic expression analysis

**Figure 3:**
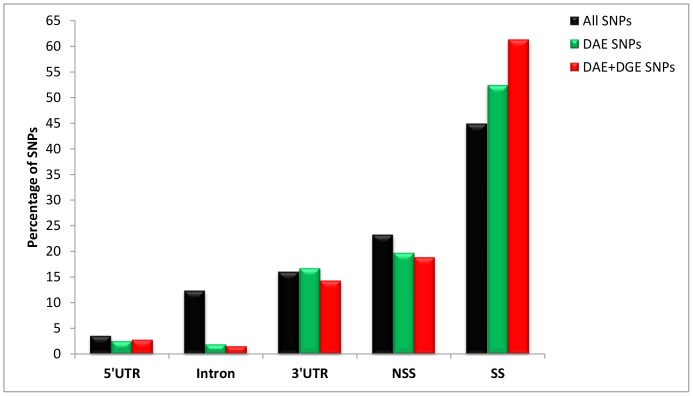
Distribution of SNPs from different regions of the *E. nitens* transcriptome. All SNPs – All SNPs identified that are common in both Florentine and Meunna; DAE SNPs – SNPs that showed differential allelic expression (Bonferonni P<0.0001) in both trials; DAE+DGE SNPs – SNPs with differential allelic expression present in genes with differential gene expression (FDR<0.05).

We studied differential allelic expression of SNPs from candidate genes to identify potential functional markers. In Meunna, 303,648 and 318,733 SNPs were identified in high and low pulp yield samples, respectively. Of these, 280,610 SNPs were present in both the samples. In Florentine, 139,408 and 149,633 SNPs were identified in high and low pulp yield samples, respectively. A total of 135,886 SNPs were common between the two samples. In total, 114,667 SNPs were common to both Meunna and Florentine. Most of these SNPs (45%) were synonymous and the remainder were non-synonymous, 3′UTR, intron and a small proportion of them were 5′UTR (Fig. 3).

To identify putatively differentially expressed alleles between low and high pulp yield samples based on allele frequency differences, a chi-square test was performed using 114,667 SNPs that are common to both the trials. Using a conservative Bonferroni corrected P value of 0.0001, we identified 27,708 and 9076 SNPs that were differentially expressed in Meunna and Florentine, respectively (Table 4, Table S4). Of these, 3390 SNPs showed DAE in both the trials and 2103 (62%) of these had allelic frequencies in the same direction in both the trials indicating the robustness of allelic expression of these SNPs. These 2103 SNPs come from 1068 unique genes (Table S4).

**Table 4:** Differential allelic expression between low and high KPY samples in two populations.

Gene ID	SNPPosition	SNPType	Meunna						Florentine						TAIR gene annotation
			High KPY			Low KPY			High KPY			Low KPY			
			Allele-A	Allele-B	Freq	Allele-A	Allele-B	Freq	Allele-A	Allele-B	Freq	Allele-A	Allele-B	Freq	
Eucgr.E01218*	13097458	5′UTR	830	917	0.52	941	290	0.24	1090	934	0.46	1821	275	0.13	acyl-CoA-binding protein 6
Eucgr.K02930*	37329597	3′UTR	2373	1057	0.31	1962	337	0.15	2469	916	0.27	3386	166	0.05	ATP binding cassette subfamily B1
Eucgr.D01413*	25222772	3′UTR	299	3254	0.92	2014	1680	0.45	893	2983	0.77	1898	1798	0.49	clone eighty-four
Eucgr.K02283*	29962223	Non-Syn	522	3116	0.86	915	1870	0.67	674	3163	0.82	1670	2176	0.57	glutamine synthase clone R1
Eucgr.I00879	18049108	Syn	2323	1410	0.38	1744	1723	0.50	3091	765	0.20	1797	1504	0.46	Granulin repeat cysteine protease family protein
Eucgr.F00715	9407802	Syn	3398	444	0.12	3067	718	0.19	2346	0	0.00	2863	788	0.22	mannose-1-phosphate guanylyltransferase (GDP)s
Eucgr.J01079*	11780983	Syn	2701	1037	0.28	1691	1317	0.44	2796	895	0.24	1394	1811	0.57	phenylalanine ammonia-lyase 2
Eucgr.J01079*	11781814	Syn	3883	55	0.01	1574	292	0.16	3108	91	0.03	1351	498	0.27	phenylalanine ammonia-lyase 2
Eucgr.H01079*	13113728	Syn	160	3701	0.96	192	1273	0.87	1	3765	1.00	460	2565	0.85	P-loop nucleoside triphosphate hydrolases superfamily protein
Eucgr.B02229*	43555157	Syn	488	2946	0.86	398	991	0.71	690	2874	0.81	1698	1692	0.50	Protein kinase superfamily protein
Eucgr.C04168	76452675	Syn	1477	2453	0.62	2424	1449	0.37	873	2234	0.72	1874	1405	0.43	RAN GTPase 3
Eucgr.F02028*	27195221	3′UTR	2739	998	0.27	1280	937	0.42	3769	181	0.05	2815	1112	0.28	RING/U-box superfamily protein
Eucgr.G01417*	24500040	Syn	2543	1367	0.35	2059	591	0.22	2950	759	0.20	3727	99	0.03	S-adenosyl-L-methionine-dependent methyltransferases superfamily protein
Eucgr.D00982*	17649968	Syn	3765	88	0.02	839	168	0.17	3372	210	0.06	1488	584	0.28	Single hybrid motif superfamily protein
Eucgr.J02983	37072540	Syn	2296	1426	0.38	2901	855	0.23	1579	2126	0.57	2673	1044	0.28	Translation machinery associated TMA7
Eucgr.D01612*	29807599	3′UTR	2151	1757	0.45	758	1267	0.63	2204	1754	0.44	681	2295	0.77	tubulin beta 8
Eucgr.F00470*	5900107	Syn	2716	1173	0.30	3483	299	0.08	2547	1399	0.35	3363	469	0.12	Tubulin/FtsZ family protein
Eucgr.F00470*	5900855	Syn	1864	1932	0.51	2246	1572	0.41	1864	1983	0.52	2871	1033	0.26	Tubulin/FtsZ family protein
Eucgr.F00119	2072476	3′UTR	662	2869	0.81	401	2974	0.88	763	2861	0.79	17	3593	1.00	Uncharacterised protein family SERF
Eucgr.F00119	2072453	3′UTR	699	2973	0.81	436	3257	0.88	647	2102	0.76	72	2613	0.97	Uncharacterised protein family SERF
	37773406	Intergenic	515	643	0.56	2300	374	0.14	136	251	0.65	2413	249	0.09	No-Hit
	21970009	Intergenic	0	1755	1.00	297	1129	0.79	3	2240	1.00	294	702	0.70	No-Hit
	22010089	Intergenic	288	3622	0.93	464	3452	0.88	11	2001	0.99	955	2890	0.75	No-Hit
Eucgr.K00671*	7466804	Intron	3329	142	0.04	2302	246	0.10	2349	409	0.15	651	1846	0.74	No-Hit
Eucgr.A01856*	28919720	3′UTR	3877	7	0.00	2384	57	0.02	3781	142	0.04	2506	690	0.22	unknown protein

Freq – Frequency of Allele-B; DGE - Differential Gene Expression; *Genes also showing DGE.

Of the 2103 SNPs showing DAE, 640 SNPs (30%) occurred in 313 unique genes that showed DGE (total gene expression). These SNPs may be the *cis*-acting regulatory variants that influence total gene expression directly or SNPs in high linkage disequilibrium with the *cis*-acting polymorphisms. In other words, 313 genes had both differential gene and differential allelic expression between low and high KPY samples. Most of the SNPs (61%) which showed DAE and DGE were synonymous SNPs (Fig. 3), suggesting a common role of synonymous SNPs as *cis*-acting variants. Genes that showed differential expression at both gene and allele levels included cellulose synthases, COBRA-like proteins, FASCICLIN-like arabinogalactan proteins, protein kinase superfamily protein, S-adenosylmethionine synthetases and beta-tubulins. Among the 640 SNPs that showed both DGE and DAE 389 SNPs were synonymous, 120 were nonsynonymous, 91 were 3′UTR, 18 were 5′UTR and 10 were intronic SNPs. These intronic SNPs may come from unspliced pre-mRNAs.

### Gene Ontology (GO) enrichment analysis of genes having differentially expressed alleles

GO enrichment analysis was conducted at two levels: 1) for genes that showed both DGE and DAE and 2) for genes that showed only DAE but no DGE. GO enrichment analysis revealed 24 categories (FDR 5%) for genes that had both DGE and DAE (Table 5). Most of these gene categories belonged to processes related to cell wall development. A total of 56 gene categories were enriched for genes that had only DAE but no DGE (Table S5). Most of these categories included genes involved in catabolic and metabolic processes (growth) and genes responding to abiotic stress factors.

**Table 5:** Gene categories enriched among genes that had both DGE and DAE.

GO Category	Total genes	DE genes	FDR
GO:0000902_cell_morphogenesis	29	4	0.04
GO:0000904_cell_morphogenesis_involved_in_differentiation	15	3	0.03
GO:0006725_cellular_aromatic_compound_metabolic_process	40	5	0.02
GO:0006886_intracellular_protein_transport	67	8	0.00
GO:0008104_protein_localization	106	10	0.00
GO:0008544_epidermis_development	22	4	0.02
GO:0009698_phenylpropanoid_metabolic_process	22	5	0.00
GO:0009699_phenylpropanoid_biosynthetic_process	17	4	0.01
GO:0009913_epidermal_cell_differentiation	21	4	0.02
GO:0015031_protein_transport	100	10	0.00
GO:0016192_vesicle-mediated_transport	45	6	0.01
GO:0019438_aromatic_compound_biosynthetic_process	27	4	0.03
GO:0019748_secondary_metabolic_process	37	5	0.02
GO:0030154_cell_differentiation	43	5	0.03
GO:0032989_cellular_component_morphogenesis	30	4	0.04
GO:0033036_macromolecule_localization	136	11	0.00
GO:0034613_cellular_protein_localization	68	8	0.00
GO:0045184_establishment_of_protein_localization	100	10	0.00
GO:0046907_intracellular_transport	88	8	0.01
GO:0048869_cellular_developmental_process	55	6	0.02
GO:0051641_cellular_localization	102	8	0.02
GO:0051649_establishment_of_localization_in_cell	95	8	0.02
GO:0070727_cellular_macromolecule_localization	71	8	0.00
GO:0071310_cellular_response_to_organic_substance	29	4	0.04

FDR - Fisher’s exact *p* value corrected for multiple comparisons.

### Genes showing selection signature based on Ka/Ks ratios

To identify the genes showing patterns of positive selection among the genes expressed in the cambial tissue we compared Ka/Ks ratios. The Ka/Ks ratios compare the number of nonsynonymous substitutions per nonsynonymous site (Ka) to number of synonymous substitutions per synonymous site (Ks) which can help identifying genes under selection. To estimate the Ka/Ks ratios we combined the sequence alignment files (BAM) from all the six bulks in each trial. In Meunna, using ‘Popoolation’ package, we identified 422,674 SNPs from within 24,068 genes. The Ka/Ks ratios among the genes ranged from 0.02 to 6.62 with a mean of 0.57 suggesting most genes are under purifying selection. Signatures of positive selection were observed for 852 genes (3.5% of total genes) that had a Ka/Ks ratio of more than 1.5. In Florentine, 218,446 SNPs from within 21,781 genes were identified. The average Ka/Ks ratio (0.50) and the range (0.015 to 7.54) are similar to Meunna. Overall, 598 genes (2.8%) had a Ka/Ks ratios of more than 1.5 suggesting the action of positive selection on these genes.

By comparing the two trials we observed in total 196 genes which had Ka/Ks ratios of more than 1.5 in both the trials strongly suggesting that these genes are under positive selection. Of these 196 genes, 27 genes also showed DGE between low and high KPY (growth) samples in both the trials (Table 6). Also, ten SNPs from seven genes that showed signatures of positive selection in both trials, showed DAE between low and high KPY (growth) samples in both the trials (Table S6). Seven of these SNPs were nonsynonymous, two were synonymous and one was within the 5′UTR. None of the genes containing these ten SNPs showed DGE in both trials suggesting these SNPs might be *trans*-acting SNPs.

**Table 6:** Genes showing signatures of positive selection and differential expression between low and high KPY samples.

Gene ID	Meunna			Florentine			TAIR gene annotation
	Ka/Ks	LogFC	FDR	Ka/Ks	LogFC	FDR	
Eucgr.J00740	1.60	−1.35	0.00	2.00	−0.99	0.01	CCCH-type zinc fingerfamily protein with RNA-binding domain
Eucgr.B00205	1.72	0.97	0.02	3.95	1.10	0.01	cytochrome P450, family 71, subfamily A, polypeptide 25
Eucgr.J00341	1.58	−1.15	0.01	1.81	−0.96	0.01	Eukaryotic aspartyl protease family protein
Eucgr.B01107	1.55	−2.16	0.00	2.04	−1.42	0.00	Glycoprotein membrane precursor GPI-anchored
Eucgr.H01694	1.77	2.00	0.00	1.70	1.68	0.02	GTP-binding protein Obg/CgtA
Eucgr.C02287	1.53	−1.31	0.00	1.52	−0.83	0.01	Integral membrane Yip1 family protein
Eucgr.E00787	6.62	1.34	0.01	3.71	1.12	0.01	Late embryogenesis abundant (LEA) hydroxyproline-rich glycoprotein family
Eucgr.H01335	3.16	1.20	0.00	2.20	0.98	0.01	Low temperature and salt responsive protein family
Eucgr.D00591	1.61	−1.80	0.00	1.60	−0.93	0.01	NAC domain containing protein 10
Eucgr.H04550	1.55	1.92	0.00	1.90	3.31	0.00	Nodulin MtN3 family protein
Eucgr.F00558	2.08	−1.64	0.01	1.68	−1.38	0.00	Pathogenesis-related thaumatin superfamily protein
Eucgr.B00466	1.71	−1.26	0.00	1.61	−0.84	0.02	Plant invertase/pectin methylesterase inhibitor superfamily protein
Eucgr.J02069	1.61	1.64	0.01	1.85	1.66	0.00	Plant protein 1589 of unknown function
Eucgr.B01716	2.48	−1.90	0.00	4.67	−1.77	0.00	Plant protein of unknown function (DUF868)
Eucgr.A02216	1.67	−3.50	0.00	1.68	−1.75	0.01	PLC-like phosphodiesterases superfamily protein
Eucgr.L01019	2.05	−2.08	0.00	1.68	−1.07	0.01	proline-rich family protein
Eucgr.G01970	2.07	3.39	0.00	2.49	4.12	0.00	related to AP2 6l
Eucgr.J02113	1.54	−0.95	0.02	1.50	−1.04	0.00	related to AP2.7
Eucgr.G02317	2.28	−1.33	0.01	1.59	−1.13	0.00	ribosomal protein L15
Eucgr.F00721	1.72	−1.18	0.01	1.50	−0.94	0.04	RNAse THREE-like protein 2
Eucgr.K01898	2.04	0.95	0.03	2.26	1.37	0.01	ubiquitin-specific protease 13
Eucgr.H04424	2.36	−1.68	0.03	2.02	−2.17	0.00	Unknown protein
Eucgr.C00838	2.80	−1.31	0.00	3.72	−1.12	0.00	Unknown protein
Eucgr.A02598	3.61	−1.38	0.00	1.56	−0.93	0.00	Unknown protein
Eucgr.E02240	1.77	1.67	0.00	1.50	1.03	0.05	Unknown protein
Eucgr.G02473	1.63	1.31	0.00	1.86	1.56	0.00	Unknown protein
Eucgr.F03994	2.16	1.22	0.02	1.72	1.68	0.00	Unknown protein

To identify the biological processes associated with genes showing selection signatures we conducted GO enrichment tests. A total of six GO categories were enriched in both trials for genes showing signatures of positive selection (Table 7). All six categories include genes involved in apoptosis, cell death and defense responses.

**Table 7:** Gene categories enriched among genes showing signatures of positive selection.

GO category	Meunna			Florentine		
	Total genes	Selected genes	FDR	Total genes	Selected genes	FDR
GO:0012501_programmed_cell_death	76	19	0	66	13	0
GO:0006915_apoptosis	68	19	0	59	12	0
GO:0008219_cell_death	83	19	0	71	13	0
GO:0016265_death	83	19	0	71	13	0
GO:0006952_defense_response	137	25	0	122	17	0
GO:0006950_response_to_stress	355	36	0	337	24	0.09

Selected genes – Genes having Ka/Ks >1.5; FDR - Fisher’s exact *p* value corrected for multiple comparisons.

## Discussion

We analysed samples from the extremes of the distribution of KPY in two *E. nitens* trials which also differed in growth. By examining whole transcriptome data we identified several genes and alleles whose expression is correlated with variation in KPY and/or growth. Most of the genes down-regulated in low KPY (low growth) samples (up-regulated in high KPY samples) were related to cell wall biosynthesis and growth. The down regulation of growth genes in low KPY samples may be due to the positive correlation observed between KPY and growth (DBH, Figure S1). Most of the up-regulated genes in low KPY and low growth samples (the down-regulated genes in high KPY samples) were involved in biotic and abiotic stress tolerance. Numerous studies, particularly in humans, have been reported in which RNA from extreme phenotypes has been sequenced to identify alleles or genes with expression correlated with the trait [29]–[31]. This is one of the first RNA-Seq studies in forest trees that exploits phenotype extremes (low and high KPY/growth) to identify differentially expressed genes and alleles potentially affecting KPY and growth. We identified several genes, including some previously uncharacterized transcripts that are differentially expressed between extreme phenotypes. The main advantage of an RNA-Seq experiment is that in addition to identifying candidate genes, polymorphisms potentially influencing the traits can also be obtained from the same data set. Accordingly, we identified a number of polymorphisms and some of these are potential functional polymorphisms that showed DAE. These variants, particularly the functional variants, can be targeted for application in many downstream analyses including association studies and genomic selection. In addition, we identified putative signatures of positive selection in several genes in this study. Comparison of results from two different trials facilitated the identification genes and SNPs that are consistently differentially expressed across environments.

### Cell wall-related genes down-regulated in low KPY samples

We identified more than 6000 (23%) and 7000 (30%) genes that are differentially expressed between low and high KPY samples in the Meunna and Florentine trials, respectively. In spite of different site conditions and time of sample collection, around 4000 genes showed consistent patterns of gene expression across both the trials, suggesting the expression of these genes is relatively stable in different environments. About 2500 genes were down-regulated in low KPY (growth) samples and most of them are related to cell wall biosynthesis and growth. These biologically relevant genes are good candidate genes for KPY and growth and other related wood traits. Genes down-regulated in low KPY (up-regulated in high KPY) samples include cell wall-related genes (cellulose synthases, PAL, SAMS, laccases, cinnamate-4-hydroxylase, COBRA-like protein, FASCICLIN-like arabinogalactan proteins, expansions, pectin-lyase like, plant invertase/pectin methylesterase inhibitor superfamily), glycosyl related genes (glycosyl hydrolase, UDP-Glycosyltransferase superfamily protein), and transcription factors (NAC, MYB). Genes that are down-regulated in low KPY samples in our study have also been found to be preferentially expressed in xylem tissues in several other studies. This includes microarray-based studies in tree species which compared different tissue types such as xylem vs phloem [32], shoot apical meristem vs mature xylem [33] and leaves vs xylem [34].

All of the genes that are up-regulated in low KPY (growth) samples belong to categories such as biotic and abiotic stress response, defense response and apoptosis. Since low KPY trees were generally smaller, this suggests that these trees experienced environmental stress, most likely due to competition effects in the trials. A transcriptome study in Arabidopsis thaliana revealed intra-specific competition resulted in activation of genes related to biotic and abiotic stresses [35]. Slow growing trees have been observed to have lower KPY in other tree species including *E. globulus* [36] and *Populus tremuloides* [37]. In a study involving *E. globulus* and *E. nitens* trees, Downes et al. [38] showed that irrigated trees had higher KPY compared to trees grown in rain-fed conditions. This suggests that trees with lower growth due to environmental factors, particularly water availability, are directing proportionally less carbon into cellulose.

### Prevalence of *cis*-acting polymorphisms

Thirty percent of SNPs with DAE (640) occurred in 313 genes that had DGE between high and low KPY trees. It is likely that some of these SNPs may be *cis*-acting regulatory variants controlling the expression of the gene in which they occur. Because there are more than one SNP from a gene in many instances, some of the SNPs in some genes will be in high linkage disequilibrium with the true *cis*-acting SNP. The remaining 1463 SNPs showed DAE but no DGE. Some of these variants may be *trans*-acting variants or coding variants in transcription factors that affect their binding affinities to target genes [39]. *Cis*-acting variants that are present within genes influence traits through their effects on gene expression while trans-acting variants affect transcript levels in target genes by interacting with *cis*-regulatory sequences [40]. While studying regulatory pathways that affect hematopoietic stem cell function using recombinant inbred mouse stains, Bystrykh et al. [41] showed strong association of the controlling locus with mRNA expression levels for *cis*-acting QTLs. In a similar study, investigating two tissues of rat recombinant inbred lines important to pathogenesis of the metabolic syndrome, Hubner et al. [42] observed 85–100% of eQTLs were regulated in *cis* in both the tissues. *Trans*-acting polymorphisms are difficult to identify compared to *cis*-acting polymorphisms for two reasons [43]. Unlike *cis* variants, *trans* variants can be anywhere in the genome relative to the target gene. Also, the effects of *trans* variants on gene expression are generally smaller than the effects produced by *cis* variants.

In this study, most of the genes (95%) that showed both DGE and DAE showed down regulation in low KPY samples at the gene level. That is, most of the cell wall-related genes had both differential total gene expression and differential allelic expression suggesting that these variants which showed DAE may be the *cis*-acting variants influencing gene expression. However, most of the growth and stress responsive genes had only differential total gene expression possibly controlled by *trans*-acting polymorphisms.

### Synonymous SNPs are not always “silent”

There was a greater tendency for synonymous, rather than non-synonymous, SNPs to be associated with genes that exhibited DAE and both DAE and DGE (see Fig. 3). This is in line with the expectation that nonsynonymous SNPs are more likely to affect phenotype by altering the amino acid structure, while synonymous SNPs are more likely to influence the trait through their effects on gene expression [6], [44]. Synonymous SNPs can affect RNA secondary structure and cause allelic imbalance that could alter the expression of a gene. For example, a synonymous SNP in the corneodesmosin gene induced allele-specific gene expression and led to increased mRNA stability in a psoriasis study across diverse ethnic groups [45]. A synonymous SNP in *EniCOBL4A* gene was associated with cellulose content by affecting allelic expression [44]. In addition to this, synonymous SNPs can also affect protein expression at the post-transcriptional level [46]. These results suggest that synonymous and other silent polymorphisms are also important in affecting the phenotype and focussing only on nonsynonymous SNPs in molecular studies will result in many functional variants being overlooked.

### Detection of signatures of positive selection at apoptosis and defense related genes

Higher Ka/Ks ratios could be due to lower constraints on nonsynonymous mutations in some genes, or through enrichment of nonsynonymous mutations by positive selection [47]. As observed in many studies, most of the genes in this study were under purifying selection based on low Ka/Ks ratios. However, 196 genes (0.9% of total genes) showed signatures of positive selection by having Ka/Ks ratios greater than 1.5. Interestingly, based on GO enrichment analysis, all the gene categories are related to apoptosis and defense response. In an *Eucalyptus camaldulensis* transcriptomics study only 2% of the genes showed signatures of positive selection and most of them are related to apoptosis and cell death [7]. These consistent results across two eucalypt species suggest that apoptosis and stress-related genes are more rapidly evolving. Apoptosis, a process of programmed cell death, is important for plant development and defense [48]. Similar results were also found in other studies. In rice, an overrepresentation of genes involved in defense response and apopstosis in eQTLs were observed [49]. Also, a study comparing the genomes of humans and chimpanzees to identify positively selected genes [50] reported an enrichment of immunity, defense and apoptosis related genes among the positively selected genes. Similarly, in fish, genes related to immune response and defense response were overrepresented in the positively selected gene list [51]. This rapid evolution of apoptosis genes could be due to the following reasons. First, many apoptosis genes may be newly evolved genes and thus still evolving rapidly under the action of natural selection. Second, because apoptosis related genes are involved in immune and defense response, these genes are rapidly evolving to adapt to new pathogens [52] as shown in the following examples. Bishop et al. [53] showed an excess of nonsynonymous compared to synonymous rates in plant class I chitinase in the genus *Arabis*. Plant chitinases confer resistance to diseases by degrading chitin, a component of fungal cell walls. Likewise, in wheat, signatures of diversifying selection were observed at the *Pm3* locus, which confers resistance to wheat powdery mildew, through an excess of nonsynonymous to synonymous nucleotide divergence [54]. The genes showing signatures of positive selection in this study could be valuable targets for selecting candidate SNPs for growth and survival traits in a range of *Eucalyptus* species as consistent results were obtained across two *Eucalyptus* species. However, results from this study need to be treated cautiously as pooled samples are used for detecting the positive selection signatures. These results need to be verified by sequencing of individual samples.

## Conclusions

By conducting RNA-Seq analysis in two trials we identified a number of candidate genes and alleles whose expression is correlated with KPY and growth traits in *E. nitens*. Most of the down-regulated genes in low KPY samples are cell wall-related genes, suggesting that the identified candidate genes are biologically relevant. A number of potential functional polymorphisms were also identified that showed DAE. We detected positive selection signatures in numerous genes that are consistent with the results from RNA-Seq study in *E. camaldulensis*. The genes and alleles identified in this study form a valuable resource for association and genomic selection studies.

## Supporting Information

Figure S1 **Correlation between Kraft Pulp Yield and Diameter at Breast Height in Meunna and Florentine.**
(TIF)

Figure S2 **Dendrogram of log2CPM in Meunna and Florentine.**
(TIF)

Figure S3 **Correlation between Log2 fold changes of 3953 differentially expressed genes in Meunna and Florentine.**
(TIF)

Table S1 **Differentially expressed transcripts between low and high KPY samples in Meunna.**
(XLSX)

Table S2 **Differentially expressed transcripts between low and high KPY samples in Florentine.**
(XLSX)

Table S3 **Differentially expressed transcripts between low and high KPY samples in both Florentine and Meunna.**
(XLSX)

Table S4 **Differential allelic expression between low and high KPY samples.**
(XLSX)

Table S5 **Gene categories enriched among genes that had only DAE.**
(XLSX)

Table S6 **Differentially expressed alleles between low and high KPY samples from genes showing signatures of positive selection.**
(XLSX)
