# Supplementary figures and images for: RNA-Seq Using Two Populations Reveals Genes and Alleles Controlling Wood Traits and Growth in Eucalyptus nitens
Source: PLoS One. 2014 Jun 26;9(6):e101104. doi: 10.1371/journal.pone.0101104 (PMC4072731; doi:10.1371/journal.pone.0101104)

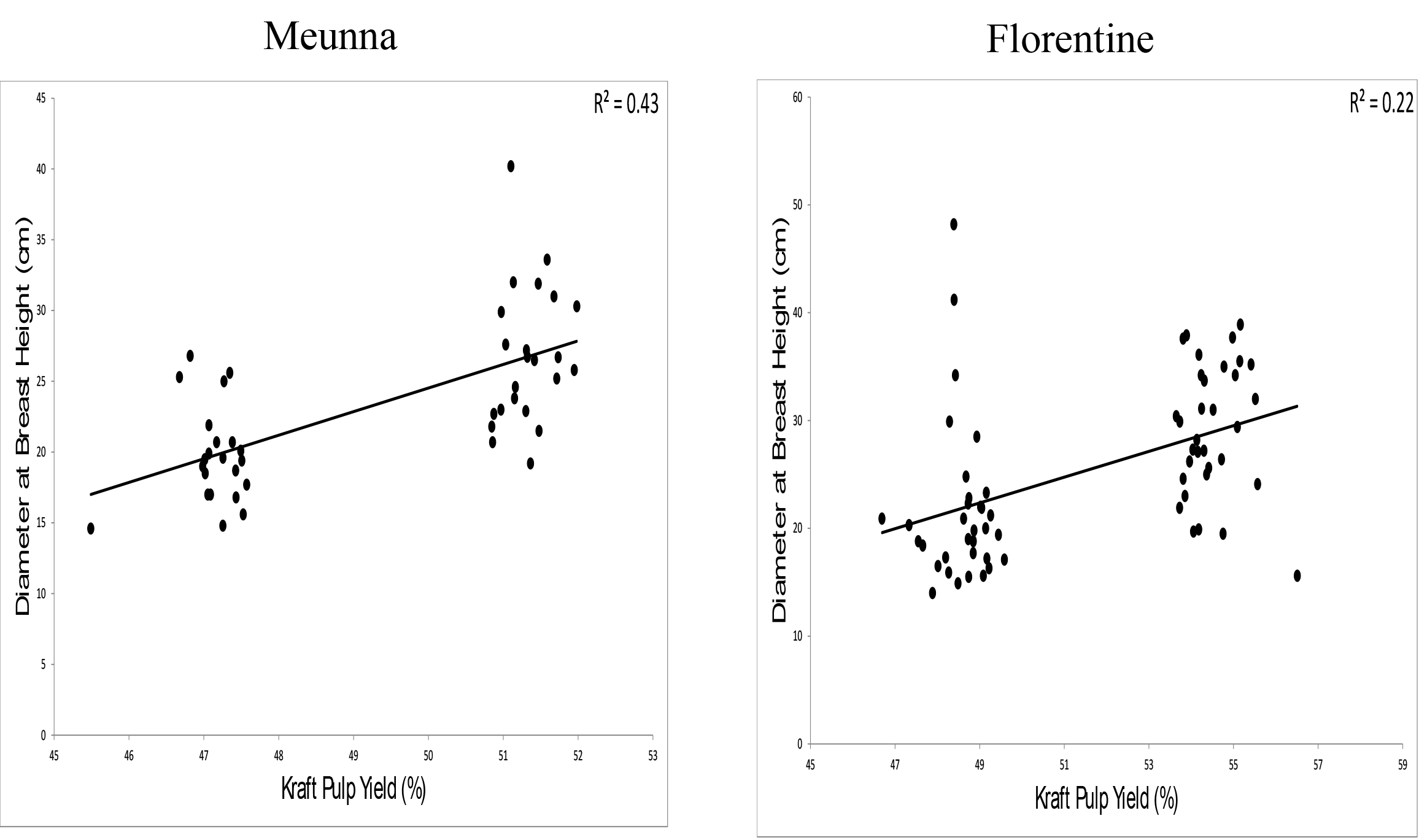

Supplement: Figure S1 — Correlation between Kraft Pulp Yield and Diameter at Breast Height in Meunna and Florentine. (TIF) [file pone.0101104.s001.tif]

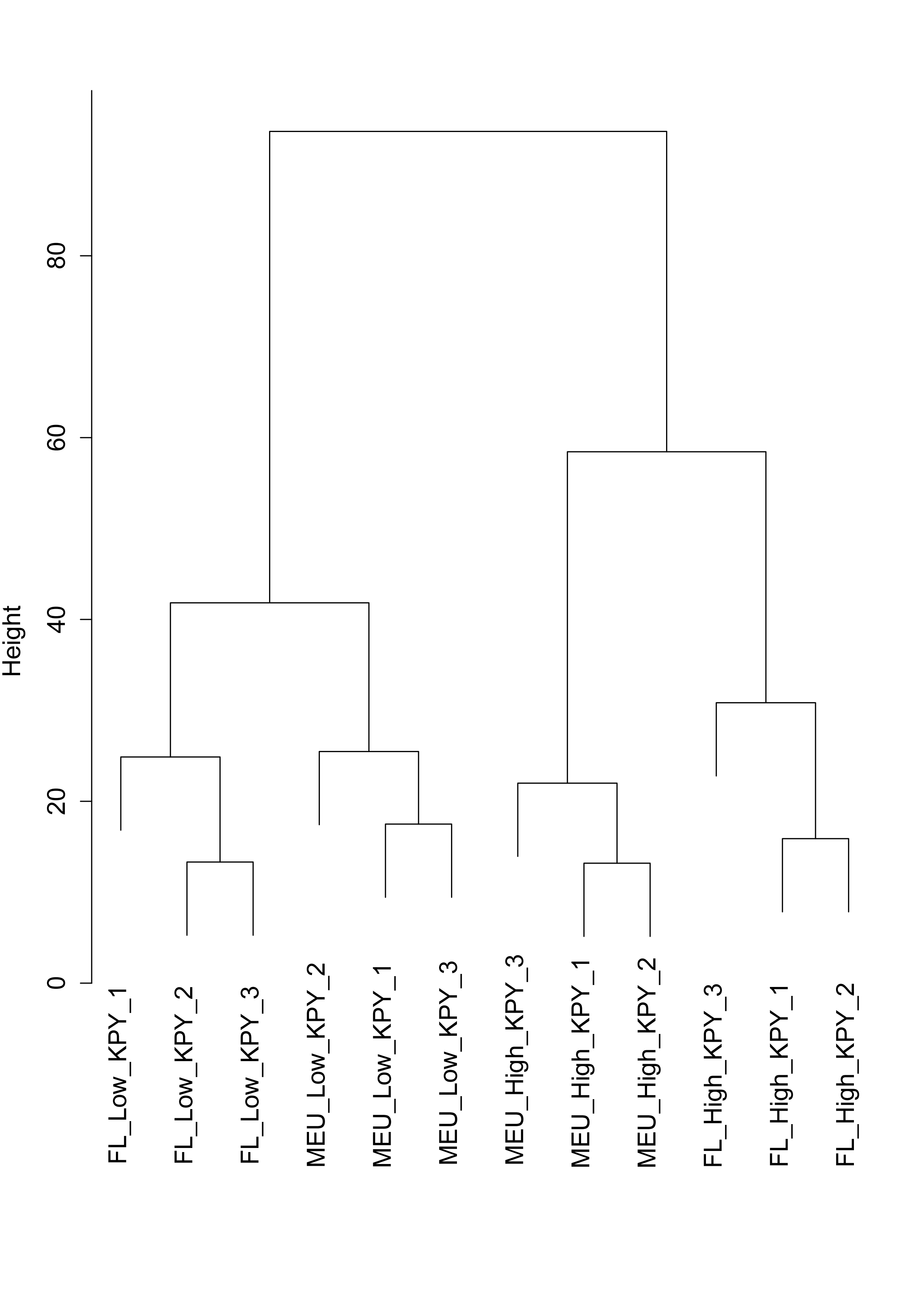

Supplement: Figure S2 — Dendrogram of log2CPM in Meunna and Florentine. (TIF) [file pone.0101104.s002.tif]

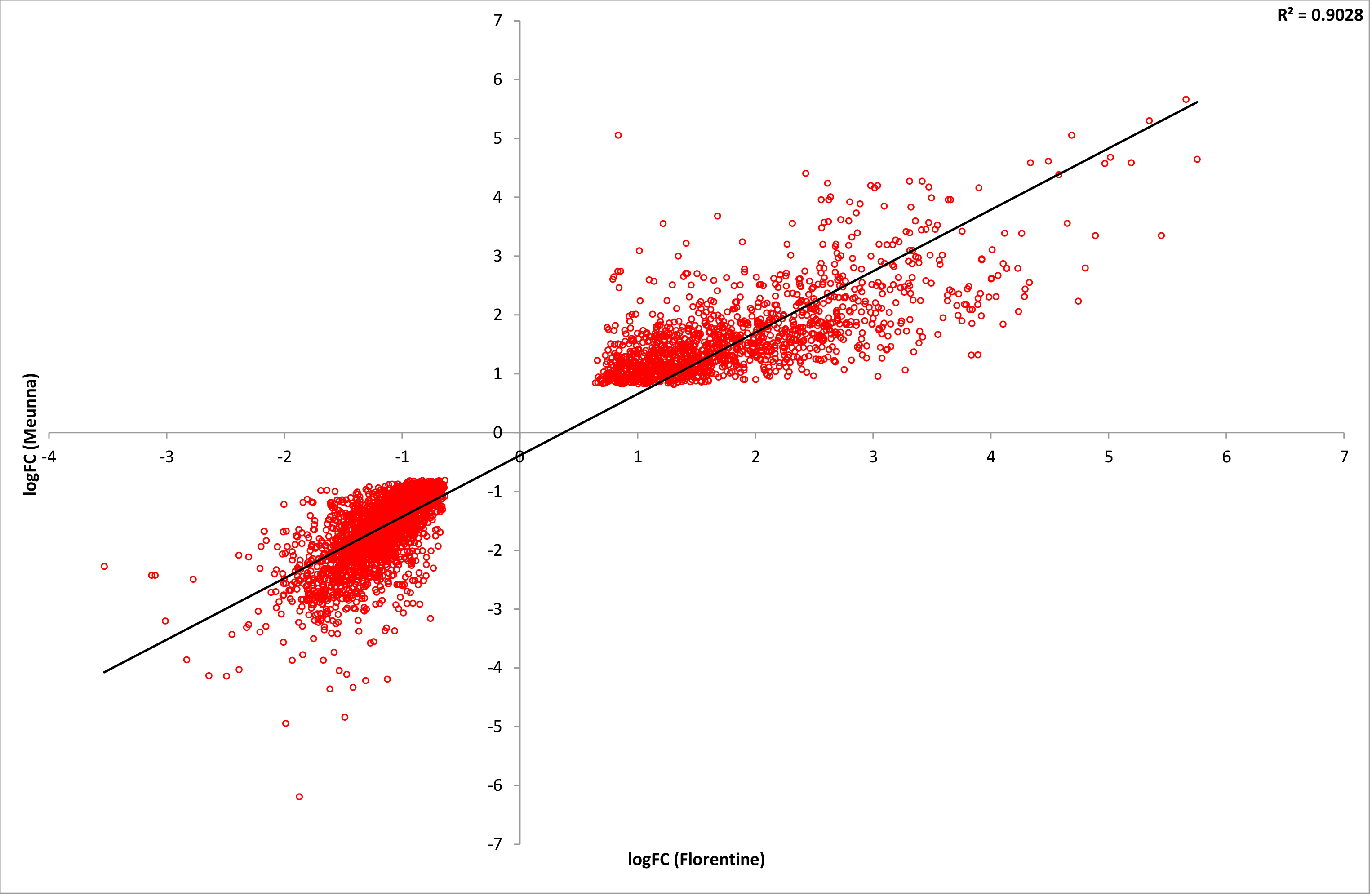

Supplement: Figure S3 — Correlation between Log2 fold changes of 3953 differentially expressed genes in Meunna and Florentine. (TIF) [file pone.0101104.s003.tif]
